# Level of Knowledge and Awareness of Female Undergraduate Students and Determinants of Knowledge of Folic Acid and Supplementation

**DOI:** 10.3390/medicina58080986

**Published:** 2022-07-25

**Authors:** Essa M. Sabi, Ahmed H. Mujamammi, Norah Alturki, Taibah Alzaid, Ateen Almutairi, Reem Algarni, Deema Almaziad, Nouf Alhumaidhi, Zeyad Kurdee, Khalid M. Sumaily

**Affiliations:** 1 Clinical Biochemistry Unit, Department of Pathology, College of Medicine, King Saud University, Riyadh 11461, Saudi Arabia; esabi@ksu.edu.sa (E.M.S.); amujamammi@ksu.edu.sa (A.H.M.); zkordee@ksu.edu.sa (Z.K.); 2 Medical Student, College of Medicine, King Saud University, Riyadh 11461, Saudi Arabia; noura.alturki0@gmail.com (N.A.); taibahalzaid@gmail.com (T.A.); ateenalmutairi@gmail.com (A.A.); reemalgarni6@gmail.com (R.A.); almaziaddeema@gmail.com (D.A.); nouf.alhumaidhi@gmail.com (N.A.)

**Keywords:** folic acid, knowledge, awareness, determinants, students

## Abstract

*Background and Objectives*: Folic acid (FA) is a necessary ingredient for numerous bodily activities including pregnancy. Because of this, women should have knowledge and awareness of the health benefits of FA supplementation. Thus, we aimed to investigate the level of knowledge on the importance of FA and determine associated factors for knowledge among female college students at King Saud University in Riyadh, Saudi Arabia. *Material and Methods*: We conducted a cross-sectional study using a questionnaire between January 2020 and February 2021 among female college students aged 17 to 26 years old. The questionnaire adapted with permission from Alnaami et al. included questions on the demographic profile of the participants as well as questions related to their knowledge and awareness of FA, FA supplementation, the importance of supplementation and their sources of knowledge of FA. *Results*: A total of 437 female undergraduate students participated in the study, 285 (65.2%) of whom were from the non-health colleges and 152 (34.8%) from the health colleges. The majority of participants were between ages 17 and 21 years old (*n* = 361, 82.6%). Half of the respondents were in their 3rd and 4th year of study (*n* = 122, 50.8%), 138 respondents (31.6%) were married, and 111 of these married women (80.4%) had children. There were 266 respondents (61.0%) who had heard and had knowledge of FA, 241 (55.3%) knew of FA timing of intake, 243 (55.7%) of FA duration of intake and 362 (83.0%) knew of the diseases prevented by FA supplementation. Linear regression analysis showed that being in the health college (B = 1.464, t = 11.37, *p* < 0.001, 95% CI = 1.211, 1.717) and a higher educational year level (B = 0.139, t = 2.442, *p* = 0.015, 95% CI = 0.027, 0.251) were the significant predictors of knowledge of FA. *Conclusions*: Knowledge of FA and FA supplementation was low at 61% considering that our study population were college students. Being enrolled in a health college and in a higher educational year level were significant positive correlates of higher knowledge of FA and FA supplementation. Despite this, there exists a gap of information regarding FA and FA supplementation particularly among single women and college educated women in the early years of their college life as well as those in non-health colleges.

## 1. Introduction

Folic acid (FA) is a necessary ingredient for numerous bodily activities, including cell division and growth, and it also acts as a cofactor in many biological reactions [1]. FA can be obtained in the form of folate from the dietary intake of a variety of foods. Additionally, fortified foods, such as whole grains and supplements, can provide FA [2].

Deficiency in FA during the first trimester is one of the leading causes of neural tube abnormalities (NTDs), such as spina bifida and other deformities [3]. As a result, women should begin taking FA supplements before conception and continue to do so for the first 12 weeks of pregnancy, as the baby may be harmed by NTDs before the woman even realizes she is pregnant [3,4,5]. To reduce the incidence of NTDs, women who are trying to conceive should consume 400 mg of folic acid every day [2]. Folic acid treatments at this dose have been demonstrated to reduce the incidence of NTDs by up to 70% in pregnant women [6,7]. In Saudi Arabia, FA deficiency during pregnancy was reported in 78.6% of cases [8].

Several studies assessed the knowledge and awareness of folic acid deficiency and supplementation among women. A survey conducted in Korea showed that 65.6% of surveyed women had heard of FA before pregnancy, and 26.4% reported on the pre-conceptional use of FA [9]. 9 In some other studies, the knowledge regarding FA need varied from 34.0% to over 67% [10,11,12,13,14,15]. However, despite their knowledge, few women, as low as 9.4% in some studies to as much as 46.3% in others, in their childbearing age take FA supplements [11,13,15]. 

Women with a university degree or higher education were found to be more likely to be aware of FA and use FA in the preconception period [9,10,13]. Other factors that were found to be associated with knowledge and awareness of FA included higher household income and being employed [12,13,14,15]. On the other hand, women aged 19–24 years, unmarried women, unemployed housewives, women with a high school diploma or lower educational level, women who had experienced unplanned pregnancy and women who had never been pregnant were likely to be aware and knowledgeable of FA or consume FA supplements [11,12,13,14,15].

Studies from Saudi Arabia showed similar results with knowledge of FA reported from 55.7% to as much as 90% [16,17,18,19]. Saudi Arabian studies also revealed that a higher level of education, specialization in health sciences and being married were factors for awareness, knowledge and use of FA [16,18]. Furthermore, a study showed that older women have the tendency to not use FA supplementation [19]. The most commonly reported source of information were healthcare professionals [13,14]. We hypothesized that a high level of knowledge of FA among college educated females exists and that the knowledge is even greater among female college students who are enrolled in health related courses. This study mainly aimed to determine the level of knowledge of FA and FA supplementation, and secondarily correlate the level of knowledge with the demographic profile among female college students at King Saud University in Riyadh, Saudi Arabia.

## 2. Subjects and Methods

We conducted a cross-sectional descriptive study using a questionnaire (Supplementary Material) among undergraduate female university students at King Saud University (KSU) in Riyadh, Saudi Arabia between January 2020 and February 2021. Female Saudi students studying for their bachelor’s degree in medicine, dentistry, pharmacy, applied medical sciences and nursing and those studying for their bachelor’s degree in humanities, business administration and science aged 17 to 26 years old were invited to participate in the survey. Students in master’s and postdoctoral programs including students aged below 17 years old and students above 26 years old, male students and students outside KSU were excluded from the study. Sample size was calculated using a 95% confidence interval and 5% margin of error, and 436 students was determined as the required sample size. 

The questionnaire used was adapted with permission from Alnaami et al. in 2018 [8]. The questionnaire included questions on the demographic profile of the participants as well as questions related to their knowledge and awareness of FA, FA supplementation, the importance of supplementation and their sources of knowledge of FA. Ethical approval for the research was granted by the Institutional Review Board (IRB) of the Deanship of Scientific Research of King Saud University. Ethical concerns regarding participants’ confidentiality and the information provided herein were trated according to the Good Clinical Practice Guidelines and the Helsinki Declaration.

Statistical analysis of the data obtained was carried out using the Statistical Package for Social Sciences (SPSS) version 25.0 (IBM-SPSS, Armonk, New York, NY, USA). Descriptive statistics were presented as mean and standard deviation for quantitative variables and as numbers and percentages for qualitative variables. Chi-square test was used to determine the relationship between categorical variables and independent samples t-test for continuous variables. The statistical relationship and association between knowledge (number of correct/yes responses to knowledge questions) and socio-demographic variables that were quantitatively coded was performed using Pearson’s correlation test. A linear regression analysis was constructed to determine the most significant factor/factors most associated with good knowledge of FA and FA supplementation. A *p* value of ≤0.05 was considered statistically significant

## 3. Results

A total of 437 female undergraduate students participated in the study, 285 (65.2%) were from non-health colleges and 152 (34.8%) were from health colleges. The mean age was 20.4 years (SD:1.9 years), with a median of 21.0 years (range: 17 to 26 years old). The majority of the participants were between ages 17 and 21 years old (*n* = 361, 82.6%). Half of the respondents were in their 3rd and 4th year of study (*n* = 222, 50.8%). There were 138 respondents (31.6%) who were married, and 111 of 138 married women (80.4%) had children. Table 1 shows the detailed socio-demographic profile of the 436 respondents.

There were 266 respondents (61.0%) who had heard of and had knowledge of FA, 241 (55.3%) knew of FA timing of intake, 243 (55.7%) of FA duration of intake and 362 (83.0%) knew of the diseases prevented by FA supplementation. However, only 44 (10.1%) of our respondents were aware of the proper FA for pregnant women. In the comparison between respondents from the health colleges versus non-health colleges students, a significant proportion of health college students knew and were aware of FA (*p* < 0.001), knew when to take FA (*p* < 0.001), knew of foods rich in FA (*p* = 0.034) and knew of diseases that can be prevented by FA supplementation (*p* < 0.001). There were no significant differences in the proportion of respondents with regard to knowledge of the duration of FA intake (*p* = 0.730) and the required dose of FA for pregnant women (*p* = 0.151). (Table 2).

With regard to year levels, the mean (SD) for respondents in year level 1 was 17.8 (0.6) years (*n* = 93, 21.3%), 19.4 (0.5) years for year level 2 (*n* = 81, 18.5%), 21.1 (0.3) years for level 3 (*n* = 120, 27.5%), 21.6 (0.9) years for level 4 (*n* = 102, 23.3%), and 23.6 (1.4) years for level 5 and above (*n* = 41, 9.4%). There were no significant difference in the proportion of respondents’ responses on the questions regarding knowledge and awareness of FA, when to take FA, foods rich in FA, duration of intake of FA and diseases that can be prevented by FA supplementation (*p* = 0.075, *p* = 0.439, *p* = 0.283, *p* = 0.700, *p* = 0.819 and *p* = 0.420, respectively. (Table 3). There was a significantly greater proportion of married women who had heard of and were aware of FA compared to single women (71.0% vs. 56.5%, *p* = 0.004). There were no significant differences in the proportion of knowledge of when to take FA, which foods are rich in FA, duration of FA intake, required dose of FA and diseases that can be prevented by FA supplementation across marital status. On the other hand, respondents who were married were significantly more aware of FA and knew the required dose for pregnant women (*p* = 0.012 and *p* = 0.018, respectively). (Table 4).

**Figure 1:**
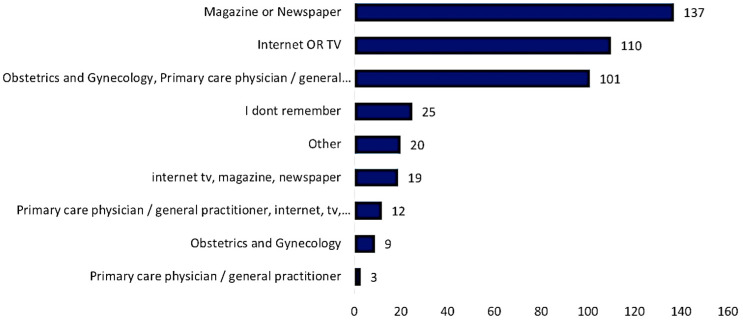
Sources of information about FA and FA supplementation.

Two hundred and thirty four respondents (53.5%) planned to take FA when they marry or during marriage. The most common source of information about FA was magazines or newspapers (*n* = 137, 31.4%) followed by the internet or television (*n* = 110, 25.2%). Information from their primary care physician or general practitioner was the primary source of information for only 3 (0.7%) of the respondents. (Figure 1).

Knowledge of FA was significantly correlated with being in one of the health colleges (*r* = 0.478, *p* < 0.001), higher year level (*r* = 0.135, *p* < 0.005), being married (*r* = 0.167, *p* < 0.001) and having children (*r* = 0.144, *p* = 0.003). Linear regression analysis showed that being in the health college (B = 1.464, t = 11.37, *p* < 0.001, 95% CI = 1.211, 1.717) and higher educational year level (B = 0.139, t = 2.442, *p* = 0.015, 95% CI = 0.027, 0.251) were the significant predictors of knowledge of FA.

## 4. Discussion

This study aimed to investigate the knowledge and awareness of FA and FA supplementation of a cohort of undergraduate female students, and determine the possible predictors for knowledge of FA and FA supplementation. In this study, we found that 61.0% of our respondents were aware and have knowledge of FA similar to the findings conducted in Korea and many other countries [9,10,11,12,13,14,15]. However, our study population were college students compared to other studies where the respondents were general populations of women. Extrapolating our results with results from previous studies, [9,10,11,12,13,14,15] our results were lower than 61% since our study population were college educated women. However, despite this, we can assume that among educated women, the knowledge of awareness of FA increases proportionately with educational level as shown in this study where many of our college students who were in their 4th to 5th and even higher educational levels possessed higher knowledge compared to those in the 1st to 3rd year levels. Multiple studies indicated that knowledge of FA is directly correlated with educational level [3,4,5,8]. However, other studies demonstrated a low level of knowledge about FA among Saudi women, even among highly educated college students [8,20]. According to a study in western Saudi Arabia, a high percentage of educated women (88%) were unaware of the importance of FA in preventing NTDs [20]. The problem is the low level of knowledge about FA among Saudi women, despite the high educational level, indicating a lack of awareness about its importance in the prevention of NTDs [8,20]. 

The positive correlation between knowledge and awareness of FA with level of education has been suggested by many authors [3,4,5,8,20,21]. Furthermore, among married women and women with children, some studies demonstrated that only a small number of these women lacked knowledge about FA and many of them possessed knowledge of some neural deformities that can be prevented by FA supplementation [22,23,24]. Similarly, our results showed that married women and women with children were significantly more aware and had more knowledge of FA compared to single women and women without children. 

Alnaami et al. conducted a study in southwestern Saudi Arabia including a total of 1366 female college students. Of these students, 806 participants were non-health care students (NHCS) and 146 were health care students (HCS). Alnaami et al. reported that a low percentage of the NHCS (29%), and a relatively higher percentage of the HCS (56%) were able to provide correct responses related to the preventive role of FA. However, the overall knowledge scores about FA were low in both groups [8]. Compared to this study, the current study results showed that 78.2% of non-health college students and 92.1% of health college students answered the question regarding the preventive role of FA correctly. The overall knowledge about FA in the study of Alnaami et al. was 38% while this study showed a higher percentage with 46%.

Similar studies concluded that a high level of knowledge about folic acid is associated with a high level of education, which may reflect in the occupation of an individual [3,22,25,26]. Another study was conducted among women in Tabuk city which showed that the major sources of information about folic acid were from gynecology clinics (62.1%) and electronic media (31%), while the current study found that 31.4% of women acquired their information from a magazine or newspaper; furthermore, the internet or TV was the second most common source of information (25.2%). This observed difference between the previously reported study and this study can readily be explained by variation in the age and marital status of the respondents [16]. 

In public health campaigns, the impact of FA administration and women’s understanding of FA and FA supplementation is mirrored by a decline in the risk of neural tube defects. Due to a lack of understanding, many women do not abide by these recommendations, and as a result, there has been very little progress made in reducing brain abnormalities in recent years. However, many nations do not opt for mandated folic acid fortification, in part due to the lack of scientific evidence supporting anticipated extra health benefits from clinical studies, physiological concerns about health dangers, and the freedom of choice issue. On the other hand, a number of publications explored the adverse effects of excessive FA supplementation during pregnancy. According to a review article, excessive FA supplementation may have a negative effect on neurodevelopmental outcomes and may affect the lipid metabolism of offspring [27,28]. It was also found that excessive FA supplementation was not linked to improved brain development in the offspring. Over supplementation, which is defined as surpassing the upper limit of the daily reference intake of FA or the recommended dietary allowance, may have detrimental consequences on human health [29]. Even though a lot of these studies used animals, further research is necessary to establish the FA dosage and intake window that are ideal for human neurodevelopment.

The small geographical area was one of the limitations of this study since it only included students from one university in Riyadh. The study design using a survey questionnaire is another limitation where response biases are very common such as whether participants provided their honest answers or not. Furthermore, the sample size of non-health college students was higher than health college students which may have tilted the selection bias towards those who were knowledgeable based on their college courses than those who were not taught some form of medicine and allied sciences. However, the strength of this study relied on our findings that not all educated women or women with higher educational levels have adequate knowledge and awareness of FA and FA supplementation. 

In order to increase the knowledge of folic acid among women of childbearing age, we recommend improved educational programs and the establishment of recommendations for health field professionals. Since the results indicated that magazines and newspapers were the primary information sources, it is critical to emphasize that this may be a useful strategy for promoting health. Despite the findings indicating a poor uptake of folic acid supplementation, it is clear that increased education is needed regarding the significance of FA during pregnancy and the negative effects of its insufficiency. Therefore, the targeted approach of information–dissemination campaigns that can be initiated by health authorities and the government should be magnified to consider all women in the childbearing age and not only women who are deficient in education and married women. We plan to undertake further studies on a larger scale involving women from all regions of the country to substantiate our arguments.

## 5. Conclusions

Knowledge of FA and FA supplementation was low at 61% considering that our study population were college students. Being enrolled in a health college and those who were in the higher educational year level were significant positive correlates of higher knowledge of FA and FA supplementation. Despite this, a gap in the knowledge regarding FA and FA supplementation was observed, particularly among single women and college educated women in the early years of their college life as well as those who were in the non-health colleges. There is a need for improved educational programs and guidelines for health professionals to raise the awareness of folic acid among women of childbearing age. Since the results showed that magazines and newspaper were the main sources of information, it is important to highlight that this could be used as an effective way to promote health. Despite the findings suggesting a low uptake of folic acid supplementation, it is apparent that greater awareness of the importance of FA during pregnancy and the consequences of its deficiency is required.

## Figures and Tables

**Table 1:** Socio-demographic profile of 436 undergraduate female students who responded to the survey.

Characteristics		Frequency (%)
Age		
	From 17–21	361 (82.6%)
	From 22–26	76 (17.4%)
Field of study		
	Health college	152 (34.8%)
	Non-health college	285 (65.2%)
Study year		
	First	93 (21.3%)
	Second	81 (18.5%)
	Third	120 (27.5%)
	Fourth	102 (23.3%)
	Fifth and above	41 (9.4%)
Marital status		
	Single	299 (68.4%)
	Married	138 (31.6%)
Have Children		
	Yes	111 (80.4%)
	No	27 (19.6%)
Number of children		
	One child	61 (54.9%)
	Two children	50 (45.1%)

**Table 2:** Comparing the correct responses on the knowledge of FA, timing of intake, duration of intake, food sources of FA, dose of FA and diseases prevented by FA between health colleges and non-health colleges students (*n* = 437).

Knowledge Questions	All Respondents *n* = 437	Health Colleges Students *n* = 152	Non-Health Colleges Students *n* = 284	*p* Values
Heard of folic acid	267 (61.1%)	132 (86.8%)	135 (47.4%)	<0.001
Knew when to take FA	242 (55.4%)	110 (72.4%)	132 (46.3%)	<0.001
Knew of food rich in FA	243 (55.6%)	95 (62.5%)	148 (51.9%)	0.034
Knew of duration of FA intake	142 (32.5%)	51 (33.6%)	91 (31.9%)	0.730
Knew the required dose of FA for pregnant women	45 (10.3%)	20 (13.2%)	25 (8.8%)	0.151
Knew of diseases that can be prevented by FA supplementation	362 (83.0%)	140 (92.1%)	222 (78.2%)	<0.001

**Table 3:** Responses to knowledge and awareness according to year levels.

Knowledge Questions	Year Levels					*p* Values
	1st *n* = 93	2nd *n* = 81	3rd *n* = 120	4th *n* = 102	5th and above *n* = 41	
Heard and aware of folic acid	58 (62.4%)	40 (49.4%)	74 (61.7%)	64 (62.7%)	31 (75.6%)	0.075
Knew when to take FA	46 (49.5%)	41 (50.6%)	73 (60.8%)	58 (56.9%)	24 (58.5%)	0.439
Knew of food rich in FA	44 (47.3%)	43 (53.1%)	71 (59.2%)	63 (61.8%)	22 (53.7%)	0.283
Knew of duration of FA intake	36 (38.7%)	26 (32.1%)	37 (30.8%)	31 (30.4%)	12 (29.3%)	0.700
Knew the required dose of FA for pregnant women	8 (8.6%)	8 (9.9%)	11 (9.2%)	12 (11.8%)	6 (14.6%)	0.819
Knew of diseases that can be prevented by FA supplementation	72 (77.4%)	70 (87.5%)	102 (85.0%)	83 (81.4%)	35 (85.4%)	0.420

**Table 4:** Proportion of responses to knowledge and awareness questions across marital status and having children.

Knowledge Questions	Single *n* = 299	Married *n* = 138	*p* Values	with Children *n* = 111	without Children *n* = 326	*p* Values
Heard and aware of folic acid	169 (56.5%)	98 (71.0%)	0.004	79 (71.2%)	188 (57.7%)	0.012
Knew when to take FA	159 (53.2%)	83 (60.1%)	0.173	68 (61.3%)	174 (53.4%)	0.149
Knew of food rich in FA	158 (52.8%)	85 (61.6%)	0.087	65 (58.6%)	178 (54.6%)	0.469
Knew of duration of FA intake	97 (32.4%)	45 (32.6%)	0.972	31 (27.9%)	111 (34.0%)	0.234
Knew the required dose of FA for pregnant women	28 (9.4%)	17 (12.3%)	0.345	18 (16.2%)	27 (8.3%)	0.018
Knew of diseases that can be prevented by FA supplementation	247 (82.9%)	115 (83.3%)	0.908	93 (83.8%)	269 (82.8%)	0.806

## Data Availability

The data that supports the findings of this study are all available in the manuscript.

## Supplementary Materials

The following supporting information can be downloaded at: https://www.mdpi.com/article/10.3390/medicina58080986/s1, Study questionnaire.
