# Supplementary material for: Level of Knowledge and Awareness of Female Undergraduate Students and Determinants of Knowledge of Folic Acid and Supplementation
Source: Medicina (Kaunas). 2022 Jul 25;58(8):986. doi: 10.3390/medicina58080986 (PMC9332624; doi:10.3390/medicina58080986)
Supplement: Supplementary file 1 [file medicina-58-00986-s001.zip › medicina-1759891-supplementary.pdf]

SUPPLIMENTARY DATA

Knowledge about the importance of folic acid among King Saud University Female Students in 2020-2021.

"Completion Of the following questionnaire will be taken as an indication of your consent to participate"

The purpose of this online survey is to estimate the level of knowledge about folic acid importance among King Saud University Female Students.

Your participation in this study is entirely voluntary and you may withdraw at any time. Any information that can be identified with you will remain confidential.

Personal information

**1. Are you a student at King Saud University in Riyadh?**(If no please submit the survey)	☐ yes	☐ No					
**2. Nationality**	☐ Saudi	☐ Non-Saudi					
**3. Age (years)**	☐ Younger than 17	☐ 17-21	☐ 22-26	☐ Older than 26			
**4. Field**	☐ Medical	☐ Science	☐ Humanities	☐ Others			
**5. Study year**	☐ 1	☐ 2	☐ 3	☐ 4	☐ 5	☐ 6	☐ 7
**6. Marital Status (If married or divorced please go to Q7, If not go to Q11)**	☐ Single	☐ Married	☐ Divorced				

Obstetric history

**7. Do you have children?**	☐ Yes	☐ No

**(If no, go to Q10)**			
**8. If yes, how many children do you have**	☐ 1	☐ 2	☐3 and more
**9. Do you have children with anomalies?**	☐ Yes	☐ No	
**10. Do you have relatives with congenital anomalies?**	☐ Yes	☐ No	

Knowledge about Folic Acid

11.	Have you heard about folic acid? (If no, please submit the survey)	☐ Yes	☐ No					
12.	Do you know when to take folic acid?	☐ Yes	☐ No					
13.	Do you know what foods are rich in folic acid?	☐ Yes	☐ No					
14.	When do you think women should take folic acid?	☐From puberty until menopause	☐In the first trimester	☐Before pregnancy	☐ mid pregnancy	☐ before labore	☐ I Don't know	
15.	The required amount of folic acid for pregnant women is:	☐ 1g	☐ 5g	☐ 10g	☐ I don't know			

16.	which of the following diseases can be prevented by folic acid intake:	☐Renal<br>☐Neurological	☐Cardiovascular<br>☐Respiratory	☐Gastrointestinal<br>☐Hematological	☐ I don't know<br>☐Other	
17.	Do you plan to take folic acid If you are married or after marriage?	☐ Yes, because I believe in its importance	☐ Yes, for precaution, even if I'm not convinced of its importance	☐ No, because many did not use it and gave birth to healthy children, those are the fate of		
18.	Which statement is the most correct in your opinion?	☐ Folic acid is important and should be taken before delivery	☐ Folic acid is important and should be taken before and during pregnancy	☐ Folic acid is important and should be taken during pregnancy	☐ I don't know	
19.	Do you have any information about spina bifida?<br>(If no, go directly to Q21)	☐ Yes	☐ No			
20.	Do you know about the relationship between spina bifida and folic acid?	☐ Yes	☐ No			
21.	your source of information is (multiple choice):	☐ Obstetrics and Gynecology	☐ Primary care physician / general practitioner	☐ I read about it in a magazine / newspaper	☐ I saw something on the Internet or TV	☐ I can't remember
